# Guominjian for allergic rhinitis: A protocol for systematic review and meta-analysis of randomized clinical trials

**DOI:** 10.1097/MD.0000000000022854

**Published:** 2020-10-30

**Authors:** Yimin Xiong, Haoran Li, Shu-Nan Zhang

**Affiliations:** a Beijing University of Chinese Medicine, Department of Clinical Medicine, Beijing; b Hospital of Chengdu University of Traditional Chinese Medicine, Chengdu, Sichuan; c China-Japan Friendship Hospital; Department of TCM Pulmonary Diseases, Beijing, China.

**Keywords:** allergic Rhinitis, guominjian, meta-analysis and systematic review, protocol

## Abstract

**Introduction:**

Allergic rhinitis (AR) is an inflammatory disease of nasal mucosa caused by IgE mediated inflammatory mediators and various immune active cells and cytokines after exposure of specific individuals to allergens. In recent years, its prevalence rate has increased gradually. Therefore, we must pay attention to carry out early intervention. However, there are still some side effects in the current drug therapy of AR, and the recurrence of AR cannot be well controlled. Some Chinese herbs have anti-allergic, anti-inflammatory and immunomodulatory effects, and have a better effect on the nasal symptoms of perennial and persistent rhinitis. The curative effect of allergic decoction on AR has been confirmed clinically. However, due to the lack of reliable evaluation means for its safety and effectiveness, it is necessary to carry out a systematic evaluation of allergic decoction in the treatment of AR, so as to lay a foundation for further research in the future.

**Methods and analysis:**

The following databases will be searched from their inception to August 2020: Electronic database includes PubMed, Embase, Cochrane Library, Web of Science, Nature, Science online, Chinese Biomedical Database WanFang, VIP medicine information, and China National Knowledge Infrastructure. Primary outcomes: nasal symptoms (sneezing, runny nose, nasal itching, and nasal congestion) and ocular symptoms (eye itching, foreign body sensation, red eyes, tearing). It can be measured by any appropriate scales or other forms of tools, such as the Total Nasal Symptom Score. Data will be extracted by 2 researchers independently, risk of bias of the meta-analysis will be evaluated based on the Cochrane Handbook for Systematic Reviews of Interventions. All data analysis will be conducted by data statistics software Review Manager V.5.3. and Stata V.12.0.

**Results:**

The results of this study will systematically evaluate the efficacy and safety of Guominjian for patients with AR.

**Conclusion:**

Through the systematic review of this study, the evidence of the treatment of AR by Guominjian has been summarized so far, so as to provide guidance for further promoting the application of Guominjian in patients with AR.

**Ethics and dissemination:**

This study is a systematic review, the outcomes are based on the published evidence, so examination and agreement by the ethics committee are not required in this study. We intend to publish the study results in a journal or conference presentations.

**Open Science Fra mework (OSF) registration number:**

September 12, 2020.osf.io/24w8n.(https://osf.io/24w8n).

## 1 Introduction

Allergic Rhinitis (AR) is a common type of Rhinitis. Epidemiological studies show that in Asia, AR affects a large number of people, ranging from 27% in South Korea to 32% in the United Arab Emirates.^[1]^ Although AR cannot be cured completely at present, the symptoms of patients can still be well controlled through standardized diagnosis and treatment, thus improving their quality of life. At present, the treatment of AR mainly includes environmental control, drug therapy, immunotherapy and surgical treatment.^[2]^ For AR medications including antihistamines (oral and nasal spray), glucocorticoid (oral and nasal spray), oral drug leukotriene, decongestants (oral and nasal spray), nasal spray resistance to choline medicine and mast cell stabilizer and so on, these drugs by acting on allergic some molecular targets of mucous membrane inflammation, blocking immune inflammatory disease caused by allergens, so it can effectively control the symptoms of AR, in the process of the clinical use of less side effects, high security. One or more may be used alone or in combination depending on the characteristics or clinical severity of the lesion. Immunotherapy, on the other hand, is a treatment method that can effectively relieve the nasal symptoms and improve the quality of life of AR patients by giving high-dose specific allergen continuous stimulation to the body and finally achieve tolerance to allergen stimulation. It can also prevent the formation of new allergens and reduce the risk of AR progressing to asthma. Some Chinese herbs have anti-allergic, anti-inflammatory and immunomodulatory effects and are safe and effective in improving the nasal symptoms of perennial and persistent rhinitis. Guominjian is a classic prescription for treating AR in the field of traditional Chinese medicine. At the same time, it can also be used for other allergic diseases.^[3]^ This prescription has a remarkable anti-wind table, the effect of dispel evils.^[4]^ Modern studies have shown that the prescription also antagonizes the itching caused by dde,^[5]^ the increased vascular permeability caused by histamine,^[6]^ lowers the IgE level in the blood,^[7]^ down-regulates the expression of PAR-2mRNA, stabilizes mast cell membranes, and inhibits mast cell degranulation.^[8]^ Although its effectiveness and safety have been proven in clinical practice, but lack of exact theoretical basis. Therefore, this study intends to evaluate the efficacy and safety of Guominjian in the treatment of AR through systematic evaluation and meta-analysis, and to explore its possible theoretical basis.

## 2 Methods and analysis

### 2.1 Study registration

The protocol has been registered in OSF(Open Science Framwork) Preregistration. September 12, 2020.osf.io/24w8n.(https://osf.io/24w8n). The protocol will follow the statement guidelines of Preferred Reporting Items for Systematic Reviews and Meta-Analyses Protocols (PRISMAP),^[9]^ Changes will be reported in the full review as required.

### 2.2 Inclusion criteria

#### 2.2.1 Type of studies

Types of studies are randomized controlled trials on investigating the efficacy and harms of Guominjian for patients with AR regardless their publication type, publication time and language. We will not consider any other studies, such as reviews, case studies.

#### 2.2.2 Types of participants

Study participants in different age ranges with AR can be included in the study without restricting nationality, sex, race, occupation or education. Patients with vasomotor rhinitis, non-AR with eosinophilia syndrome, infectious rhinitis, hormonal rhinitis, drug-induced rhinitis, aspirin intolerance triad, cerebrospinal rhinorrhea were excluded.

#### 2.2.3 Type of interventions

We will accept any forms of Guominjian as an interventional treatment in the experimental group. However, we will remove studies with combination of Guominjian and other modalities. In the control group, we accept any treatments, except any types of Guominjian, including its single or combination modes.

#### 2.2.4 Type of outcomes

The primary outcome is total nasal symptoms. It consists of nasal symptoms (sneezing, runny nose, nasal itching, and nasal congestion) and ocular symptoms (eye itching, foreign body sensation, red eyes, tearing). It can be measured by any appropriate scales or other forms of tools, such as the Total Nasal Symptom Score.

The secondary outcomes are quality of life (as identified by any scores, such as the Rhinoconjunctivitis Quality of Life Questionnaire), global non-nasal symptoms (as assessed by any validated daily or weekly diaries or scores, such as visual analogue scales), use of conventional medication (as evaluated by Medication Quantification Scale or any other scales), laboratory indicators, and any expected or unexpected adverse events.

## 3 Data sources

### 3.1 Electronic searches

The following data bases will be searched to identify eligible studies: PubMed, Embase, Cochrane Library, Web of Science, Nature, Science on line, Chinese Biomedical Database WanFang, VIP medicine information, and China National Knowledge Infrastructure. The time range is: the starting time is determined according to the first literature available, and the deadline is August 2020.

### 3.2 Other search resources

**Figure 1:**
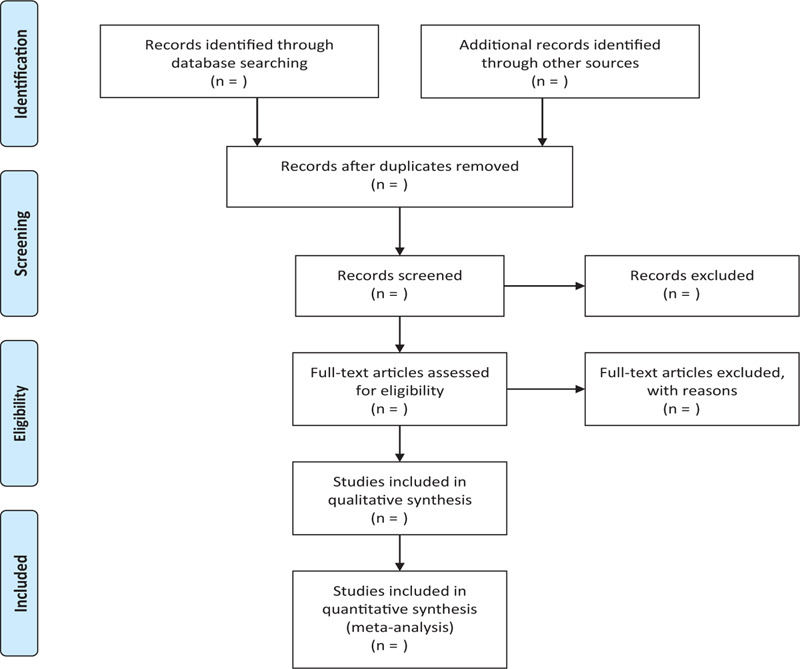
The research flowchart. This figure shows the Identification, Screening, Eligibility and Included when we searching articles.

In order to get more complete evidence, we will also retrieve other related documents by manually, such as medical textbooks, clinical laboratory manuals and so on. If it is necessary we will contact with trail author to obtain the latest clinical data. Moreover, studies associated with the review will be identified via evaluating related conference proceedings. The research flowchart is shown in Figure 1.

### 3.3 Search strategy

The following search terms will be used: randomized controlled trial/ randomized controlled trial; AR/Affection of AR; traditional chinese medicine/TCM; Guominjian/Guominjian therapy/Guominjian treatment. different retrieval strategies in Chinese and foreign databases will be used. Language restrictions are Chinese and English. There is no publication restriction. Here we take the search strategy in PubMed as an example and list in Table 1.

**Table 1:**
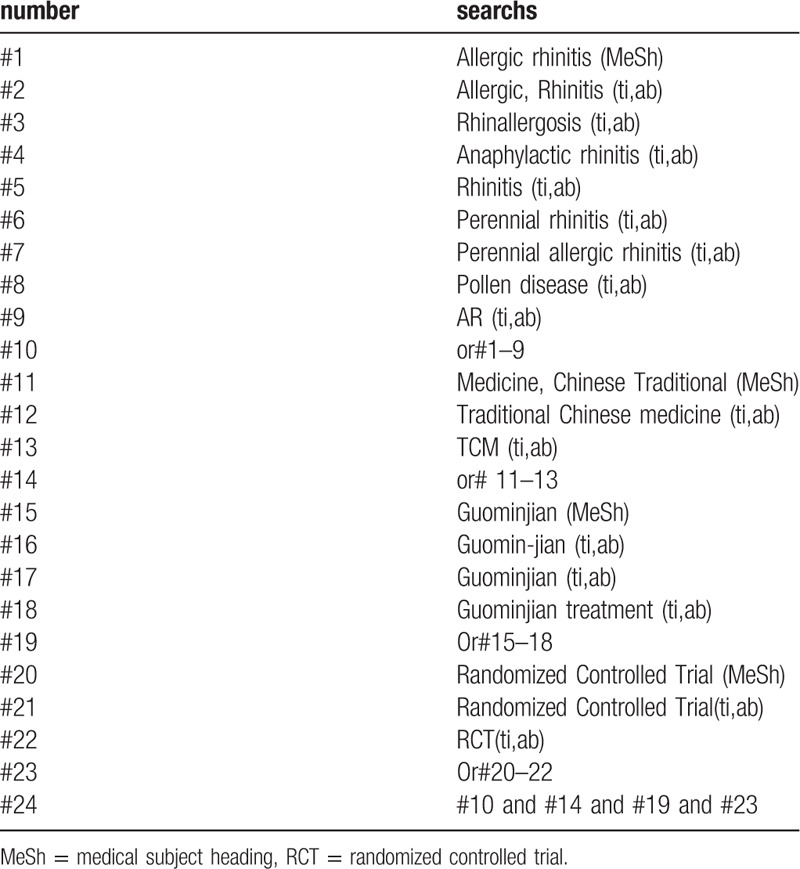
Search stragtegy sample of PubMed.

## 4 Data collection and analysis

### 4.1 Study selection

All articles in the search results were independently evaluated by 2 independent researchers (YX, HL) according to inclusion and exclusion criteria. Reviewers will then independently extract and collect the data included in the study using pre-designed data collection forms. Discrepancies will be discussedand resolved by consensus with a third author (SZ).

### 4.2 Data extraction and management

The following informations will be extracted from each study:

(1) Normal test characteristics: title, author, year.
(2) baseline data: sample size, age, gender, diagnosticcriteria, course of disease.
(3) interventions: acupuncture therapy, control of intervention details, intervention. If the information is not enough, we will contact experts and authors in this field to get relevant information.

### 4.3 Assessment of the reporting quality and risk of bias

The risk of bias will be assessed by 2 independent authors (YX and HL), together with completing the STRICTA checklist.^[10]^ The Cochrane System Evaluator's Manual give the evaluation criteria for authors to evaluated the randomized controlled trials quality. Assessing the risk of bias:

(1) random sequence generation;
(2) allocation concealment;
(3) blinding of participants and personnel;
(4) blinding of outcome assessment;
(5) incomplete outcome data;
(6) selective outcome reporting;
(7) other bias.

Any disagreement will be discussed or consulted with a third reviewer. Each them will be described from 3 levels: “high risk,” “low risk” and “unclear.”

### 4.4 Measures of a treatment effect

The dichotomous outcomes will be expressed by the Odds ratio(ORs), while the continuous data will use the Standardized mean difference(SMD). All these outcomes report 95% confidence intervals.

### 4.5 Management of missing data

We will take the method of contacting corresponding authors to obtain the missing data. If there is no response, we will analyze only the available data and describe the reason and impact of this exclusion in the paper.

### 4.6 Assessment of a reporting bias

The bias of publication will be explored through funnel plot analysis. If the funnel plot show asymmetry, it will be evaluated via the Egger and Beg tests, and P value <.05 means the publication bias is significant.

### 4.7 Assessment of heterogeneity

There are 2 main methods for testing heterogeneity, namely graphical method (funnel plot, forest plot) and statistical test (*Q* value statistic test, *I*^2^ statistic test, *H* statistic test). The *I*^2^ statistic test method shows us When *I*^2^ is 0, it means that studies are completely homogeneous, If *I*^2^>50%, it indicates there is heterogeneity in studies.

### 4.8 Data synthesis and grading of quality of evidence

The results of the study will be analyzed by RevMan 5.0 software provided by Cochrane collaborate on network. The binary data will be expressed by the odds ratio, while the continuous data will use the mean difference (MD). To test the heterogeneity of the research results, when the *I*^2^ < 50% or *P* > .1, the heterogeneity is significant. The random effect model was used for the meta-analysis, otherwise, we choose the fixed effect model.

### 4.9 Subgroup analysis

We will undertake subgroup analysis based on the different types of interventions and controls, characteristics of study or patient, and different outcome measurements.

### 4.10 Sensitivity analysis

Sensitivity analysis can not only assess the stability and reliability of the conclusions of the Meta analysis, but also assess whether the changes in the results are related to the impact of a single study. If the stability of the conclusion is poor, we can achieve When the heterogeneity test results are heterogeneous, we need to clarify the source of the heterogeneity by subgroup analysis. The effects of different types of therapy including design scheme, severity of illness, age, sex, andmild or severe AR were analyzed. We will also delete low-quality and/or medium-quality studies to check the robustness of theresults the purpose of increasing stability by changing the analysis model, inclusion and exclusion criteria, or excluding a certain type of literature.

### 4.11 Ethics and dissemination

We will publish the system review results in peer-reviewed journals, disseminated in meetings or in peer-reviewed publications. Aggregated published data will be used to exclud data of individuals, so there is no need for obtaining the ethical approval or patients’ informed consent.

## 5 Discussion

AR is a kind of caused by specific individual after exposure to an allergen mediated by IgE release of inflammatory mediators and the activity of a variety of immune cells and cytokines involved in the occurrence of inflammation in nasal mucosa disease, the main clinical symptoms include nasal itching, sneezing, flow stuff, nasal congestion, in addition to the nasal symptoms, some patients can merge bronchial asthma, resulting in asthma, cough, shortness of breath, chest tightness and other symptoms, lung clinically AR often interact with asthma and its influence, endangering human health. Some patients may have itchy eyes, tears, red eyes and burning eyes and other eye symptoms. There is a certain theoretical basis for the clinical efficacy of AR decoction. Study has shown that both low dose and high dose of allergic decoction can significantly alleviate the allergic reactions in model mice.^[11]^ In addition, a number of studies have also shown that allergy decoction can significantly improve inflammatory factors in antagonists,^[5–8]^ effectively improve patients’ allergic symptoms, and thus improve their quality of life.

In recent years, clinical experience has proved that Guominjian is an effective and safe way to treat AR. Relevant studies have proposed its possible mechanism of action, but the mechanism is still unclear, and most of the literature has not clearly pointed out the mechanism of Guominjian's treatment of AR. Therefore, it is very necessary to carry out standardized and unified experimental design in clinical studies to evaluate the efficacy, which can not only guide the application of Guominjian in AR, but also explore the mechanism of Guominjian's action in the treatment of AR.

In summary, systematic review and meta-analysis are helpful to determine the application of Guominjian in the treatment of AR, and reduce the waste of medical resources, patients’ time and money. This study can not only provide a basis for the introduction of treatment guidelines for AR, but also promote the application of Guominjian treatment to benefit more patients.

## Acknowledgment

We are grateful to Dr Zhang for technical support and advice.

## Author contributions

**Conceptualization:** Yi-min Xiong; Haoran Li; Shu-nan Zhang.

**Data curation:** Yi-min Xiong; Haoran Li.

**Formal analysis:** Yi-min Xiong; Haoran Li.

**Methodology:** Yi-min Xiong; Haoran Li; Shu-nan Zhang.

**Project administration:** Shu-nan Zhang.

**Resources:** Yi-min Xiong; Haoran Li.

**Software:** Yi-min Xiong; Haoran Li.

**Supervision:** Shu-nan Zhang.

**Writing – original draft:** Yi-min Xiong.

**Writing – review & editing:** Shu-nan Zhang.
